# How Hepatitis D Virus Can Hinder the Control of Hepatitis B Virus

**DOI:** 10.1371/journal.pone.0005247

**Published:** 2009-04-21

**Authors:** Maria Xiridou, Barbara Borkent-Raven, Joost Hulshof, Jacco Wallinga

**Affiliations:** 1 Centre for Infectious Diseases Control, National Institute of Public Health and the Environment, Bilthoven, The Netherlands; 2 Department of Mathematics, VU University Amsterdam, Amsterdam, The Netherlands; 3 Julius Center for Health Research and Primary Care, University Medical Centre Utrecht, Utrecht, The Netherlands; University of Zurich, Switzerland

## Abstract

**Background:**

Hepatitis D (or hepatitis delta) virus is a defective virus that relies on hepatitis B virus (HBV) for transmission; infection with hepatitis D can occur only as coinfection with HBV or superinfection of an existing HBV infection. Because of the bond between the two viruses, control measures for HBV may have also affected the spread of hepatitis D, as evidenced by the decline of hepatitis D in recent years. Since the presence of hepatitis D is associated with suppressed HBV replication and possibly infectivity, it is reasonable to speculate that hepatitis D may facilitate the control of HBV.

**Methodology and Principal Findings:**

We introduced a mathematical model for the transmission of HBV and hepatitis D, where individuals with dual HBV and hepatitis D infection transmit both viruses. We calculated the reproduction numbers of single HBV infections and dual HBV and hepatitis D infections and examined the endemic prevalences of the two viruses. The results show that hepatitis D virus modulates not only the severity of the HBV epidemic, but also the impact of interventions for HBV. Surprisingly we find that the presence of hepatitis D virus may hamper the eradication of HBV. Interventions that aim to reduce the basic reproduction number of HBV below one may not be sufficient to eradicate the virus, as control of HBV depends also on the reproduction numbers of dual infections.

**Conclusions and Significance:**

For populations where hepatitis D is endemic, plans for control programs ignoring the presence of hepatitis D may underestimate the HBV epidemic and produce overoptimistic results. The current HBV surveillance should be augmented with monitoring of hepatitis D, in order to improve accuracy of the monitoring and the efficacy of control measures.

## Introduction

Hepatitis D (or hepatitis delta) virus is a defective virus that requires helper functions from hepatitis B virus (HBV) for virion assembly and propagation [1]. Therefore, infection with hepatitis D can occur only with an associated HBV infection. This can happen as coinfection (infection with both viruses at the same time) or superinfection (where an already HBV-infected individual can be infected with hepatitis D); individuals coinfected or superinfected transmit both viruses [2]. Transmission routes for hepatitis D are similar to those for HBV, namely bloodborne and sexual, percutaneous, permucosal, and perinatal. Superinfection with hepatitis D is associated with higher progression rate to chronic disease and to serious complications [3], [2] and may result in suppression of HBV replication, such that an individual with dual infection transmits HBV less than an individual infected only with HBV [4], [5].

High prevalences of hepatitis D have been reported among individuals infected with HBV, but recent reports indicate that hepatitis D prevalence is on the decline. For instance, in 1986, dual infection with both viruses was found in 91% of Taiwanese drug users infected with HBV [6]; this percentage was reduced to 39% in 1997 [6]. In Italy the prevalence of hepatitis D among HBV-infected individuals declined from 23% in 1987 to 8% in 1997 [7]. It is believed that the reductions in hepatitis D are largely due to the reductions in HBV [7], as result of the introduction of HBV vaccination and risk-reduction measures taken against the spread of HIV: the decreased circulation of HBV decreases the reservoir needed for the spread of hepatitis D, thus depriving the defective virus of susceptible hosts to infect. Inversely, since hepatitis D reduces the infectivity of HBV in those dually infected, we could speculate that hepatitis D may facilitate the control of HBV.

To investigate how hepatitis D affects the HBV epidemic, we use a mathematical model describing the spread of the two viruses in a population. We show that hepatitis D prevalence is very sensitive to changes in the infectivity of HBV. We also show that if hepatitis D virus is highly transmissible, the presence of hepatitis D can result in more severe HBV epidemic.

## Methods

**Figure 1:**
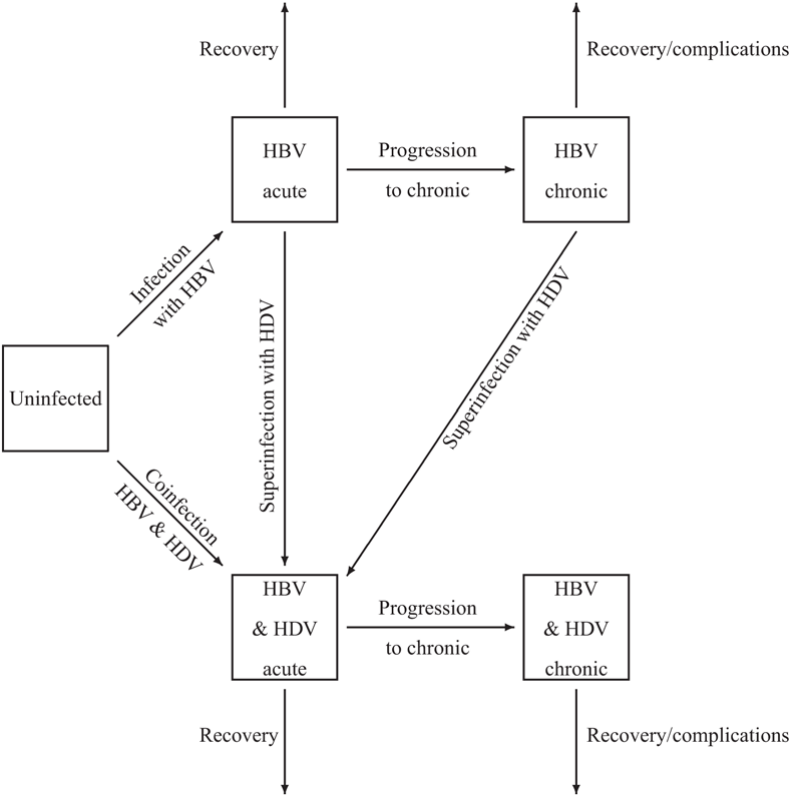
Model for the transmission of hepatitis B virus (HBV) and hepatitis D virus (HDV).

For the transmission of HBV and hepatitis D, we use a mathematical model, shown in Fig. 1. Infection with hepatitis D occurs only together with infection with HBV, as superinfection of an existing HBV infection or coinfection with both viruses at the same time. The infection is divided into two stages, a short acute stage (stage 1) and a long chronic stage (stage 2) with lower infectivity than the acute stage. Hepatitis D superinfection of an individual already infected with HBV causes a generally severe acute hepatitis with short incubation that usually leads to chronic hepatitis [2]. Therefore, in the model those with acute or chronic HBV infection who are superinfected with hepatitis D go again through the acute phase (of dual infection). Let 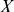 be the number of uninfected individuals, 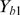 and 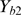 the numbers of persons infected only with HBV at stage 1 and 2, respectively, and 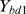 and 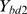 the numbers of those infected with both viruses. Let 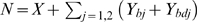 be the total population size. The model is described by the following differential equations:
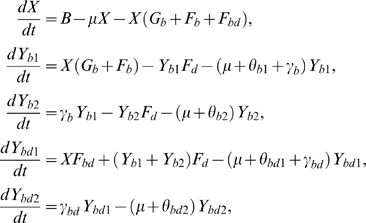
(1)where we define the per capita risks to get

- infected only with HBV from an individual with single HBV: 
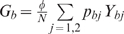
,
- infected only with HBV from an individual with dual infection: 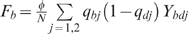,
- superinfected with hepatitis D: 
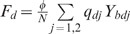
,
- coinfected with both HBV and hepatitis D: 
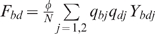
.

The definitions of the parameters are summarised in Table 1. The transmission probability of HBV from a person infected only with HBV is 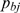, for stage *j* = 1, 2. Persons infected with both viruses in the *j*-th stage of infection, (i) transmit only HBV with probability 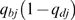, (ii) transmit both HBV and hepatitis D with probability 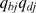, and (iii) superinfect those infected only with HBV with probability 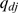. Here it is assumed that hepatitis D may affect the replication of HBV in individuals with dual infection, such that those dually infected transmit HBV less than individuals infected only with HBV [4], [5]. With *i* = *b* for single HBV infection and *i* = *bd* for dual infection, 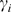 is the progression rate from stage 1 to stage 2, 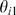 and 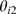 are the progression rates out of stage 1 and stage 2 (recovery or extra death due to the disease). Also, *ϕ* is the rate of partner change, *μ* is the per capita removal rate out of the population, and *B* is the rate at which new individuals enter the population.

**Table 1:** Parameter definitions and values.

Symbol	Definition	Value	Source*
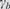	Progression rate from acute to carrier for HBV	0.4/person/year	[27]–[29]
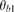	Recovery rate from acute infection with HBV	3.6/person/year	[27]–[29]
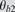	Recovery rate from chronic infection with HBV	0.02/person/year	[25], [28]
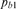	Transmission risk of HBV from person with HBV only, stage 1	0.46	[25], [30]
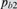	Transmission risk of HBV from person with HBV only, stage 2	0.65 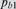	[25], [30]
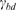	Progression rate from acute to carrier for those dually infected	2/person/year	[2], [28], [31]
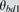	Recovery rate from acute infection for those dually infected	2/person/year	[2], [28], [31]
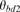	Recovery rate from chronic infection for those dually infected	0.02/person/year	[25], [28]
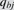	Transmission risk of HBV from person with dual infection, stage *j* = 1, 2	0.71 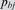	[6]
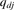	Transmission risk of hepatitis D from person with dual infection, stage *j* = 1, 2	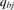	
*n*	Initial total population size	26000	[32]
*μ*	Rate of departing from the population	0.018/year	
*B*	Rate at which individuals enter the uninfected population	*μn*	
*ϕ*	Rate of partner change	1.64 partners/year	[33], [34]

HBV, hepatitis B virus. Dually infected are individuals infected with both hepatitis B and hepatitis D viruses. The transmission risks are expressed as probabilities of transmission per partnership. The stages 1 and 2 of infection (single HBV and dual) are the acute and the chronic stages, respectively.

* See details in the Supporting Information, text S1.

## Results

### Conditions for the eradication or persistence of the viruses

To investigate the long-term dynamics of the two viruses, we performed an equilibrium analysis of the system equations. This analysis allows us to find the steady state of the system and the conditions for the eradication or persistence of the viruses. Solving the model equations (1) with the left-hand side equal to zero, we find all the possible steady states. The model has three types of steady states: one where both viruses are eradicated (the disease-free equilibrium), one where only HBV remains endemic but hepatitis D is eradicated, and one where the prevalences of both viruses are non-zero (in the following it will be shown that there may be more than one point of this third type, with negative or complex entries).

#### The reproduction numbers

Further, we found conditions for the stability of the disease-free equilibrium and of the endemic equilibria (see Supporting Information, Text S1, for details). These correspond to conditions for the eradication or the persistence of the viruses, respectively, and are expressed in terms of the reproduction numbers. The *basic reproduction number* of HBV (denoted *R_b_*) is the number of secondary infections caused by an individual with HBV throughout his infectious period, if introduced in a population of uninfecteds. For this model, the reproduction number of HBV is
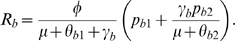
Similarly, we define the *basic reproduction number* of dual infections as the number of secondary dual infections caused by an individual with dual infection throughout his infectious period, if introduced in a population of uninfecteds:
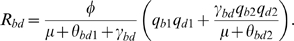
Finally, the *invasion reproduction number* of dual infections, 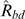, gives the number of secondary cases of dual infections that an individual with dual infection can produce throughout his infectious period, if introduced in a population where HBV is at its endemic equilibrium (see, e.g., [8]). This number is a threshold that determines whether dual infection can invade the equilibrium with only HBV and is given by
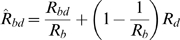
(see Text S1 for details of the calculations), where we used the notation
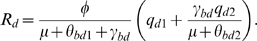



#### Stability of equilibria

**Figure 2:**
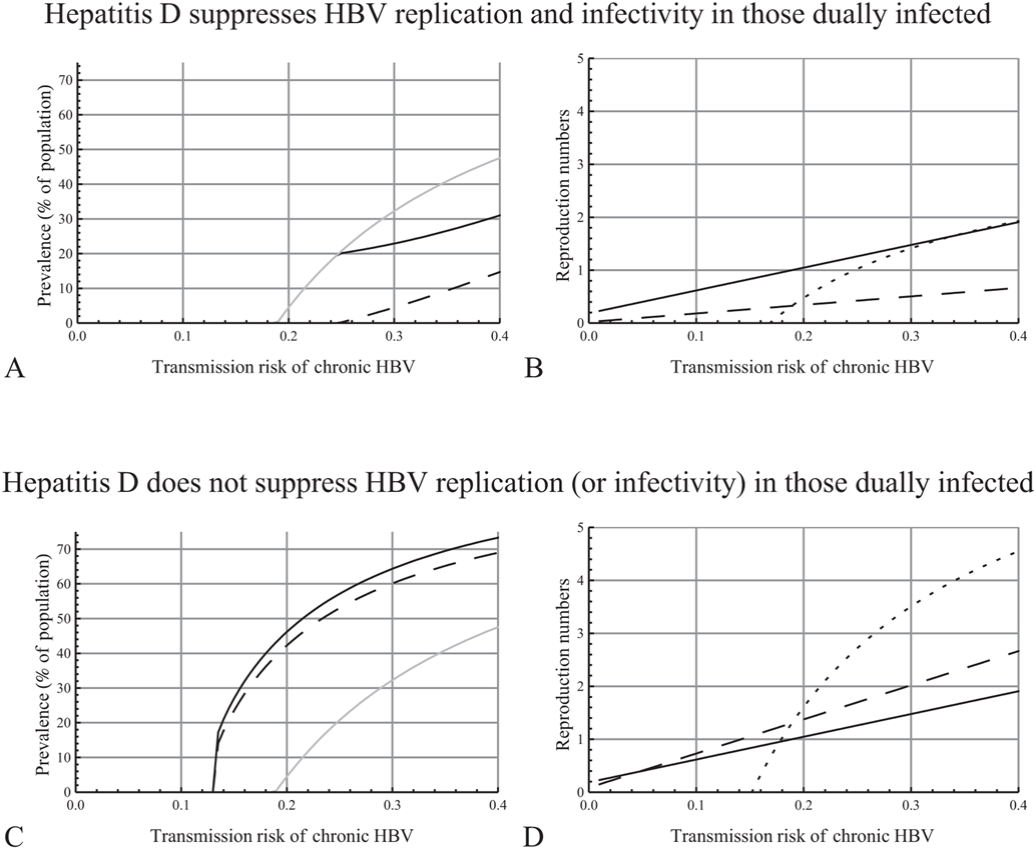
The impact of measures changing the infectivity of chronic hepatitis B virus (HBV). The transmission probability of chronic HBV from individuals with single HBV infection (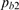) was varied from 0 to 0.4. A, B: hepatitis D reduces HBV infectivity by 50% in individuals dually infected (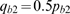); C, D: hepatitis D does not suppress HBV replication (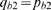). Left panels show the total prevalence of HBV (black solid line), the prevalence of hepatitis D (black dashed line), and the prevalence of HBV in a population without hepatitis D (grey line). Right panels show the reproduction numbers *R_b_* (solid line), *R_bd_* (dashed line), and 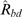 (dotted line).

**Figure 3:**
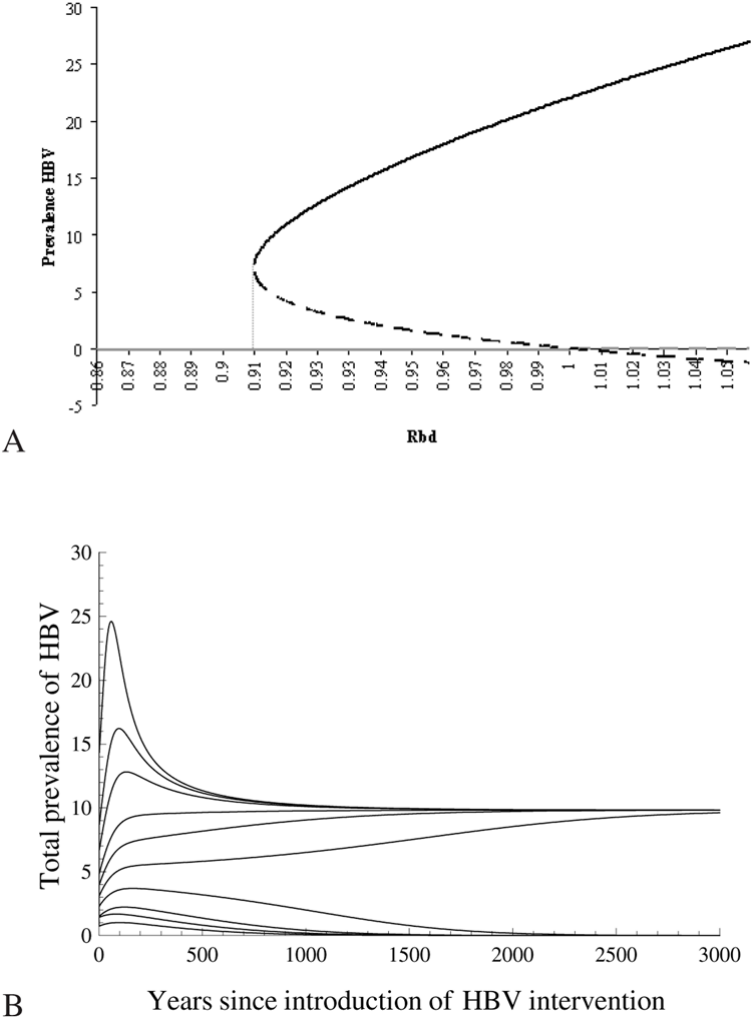
Bistability: coexistence of endemic and disease-free equilibria. A. Bifurcation diagram. The two positive equilibria are shown with black, the disease-free equilibrium with grey; solid lines correspond to locally asymptotically stable equilibria, dotted lines to unstable equilibria. Results shown here are with 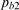 varied from 0.12 to 0.15 (*R_bd_* from 0.86 to 1.05), 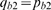, 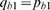, and the other parameters as in Table 1. B. The prevalence of hepatitis B virus (HBV) with different initial conditions. In all curves shown, 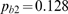, 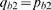, 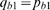, and the other parameters as in Table 1. The two positive endemic equilibria are at 9.83 (locally stable) and 4.88 (unstable).

If both basic reproduction numbers 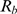 and 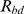 are less than one, the disease-free equilibrium is locally asymptotically stable and otherwise it is unstable. If 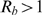 and 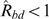, then the endemic equilibrium with only HBV (hepatitis D is eradicated) is locally asymptotically stable and unstable otherwise. For the equilibrium with both viruses present, it was not possible to find analytic conditions for its stability, due to the complexity of the system. However, we solved numerically the model equations for several combinations of the parameter values and the numerical results suggest that the endemic equilibrium with both HBV and hepatitis D is stable if either 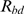 or 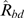 is greater than one (numerical calculations were done using Mathematica, version 6.1). This implies that even if 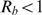, HBV may not be eradicated if 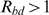. (Notice that it is not possible to have 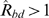 while both 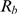 and 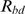 are less than one, because if 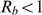 then 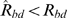.) Figures 2 and 3 show that indeed this is possible. The prevalences of the two viruses are shown in Fig. 2A,C and the reproduction numbers in Fig. 2B,D for different levels of infectivity during chronic HBV (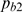 is varied from 0.01 to 0.4). If hepatitis D suppresses HBV replication (Fig.2A,B), then HBV is eradicated when 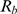 is reduced below one. However, if hepatitis D does not suppress HBV replication (Fig. 2C,D), then reducing 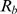 below one is not sufficient to eradicate HBV; the reproduction number of dual infections, 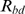, has to be also reduced below one.

#### Bistability: coexistence of endemic and disease-free equilibrium

The condition that all reproduction numbers are below one is necessary, but not sufficient for the eradication of the viruses, as shown in Fig. 3. Here we solved numerically the model equations (1) with the left-hand side equal to zero and calculated all the equilibrium points of the system, varying the transmission risk from HBV carriers as in Fig. 2C,D (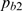 varied from 0.01 to 0.4 and 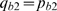, 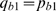). With these values the system has five equilibrium points: the disease-free equilibrium, an endemic equilibrium with only HBV, and three solutions with non-zero prevalences for both HBV and hepatitis D. Of these last three points, at least one is negative or complex for every value of 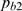 examined here, while two are positive for 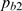 between 0.127 and 0.142. In this interval, 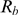 increases from 0.734 to 0.797, 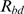 from 0.906 to 1, and 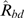 is negative. Also, with these values of 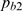, one of the positive points is locally asymptotically stable and the other unstable, while the disease free equilibrium is also locally stable (Fig. 3A; the two negative solutions and the complex values are not shown). This suggests that the model exhibits backward bifurcation, which means that an endemic and the disease-free equilibria are both stable in an area where the associated reproduction number is less than one. When 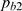 exceeds 0.142, 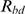 becomes larger than one, and one of the two positive points becomes negative. Fig. 3B shows HBV prevalence with 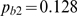, starting with different initial conditions: with the same parameter values, the system converges to an endemic equilibrium (HBV prevalence 9.83%) or to the disease-free equilibrium, depending on the initial HBV prevalence.

### How hepatitis D affects the spread of HBV

#### Variations in HBV prevalence according to hepatitis D infectivity

**Figure 4:**
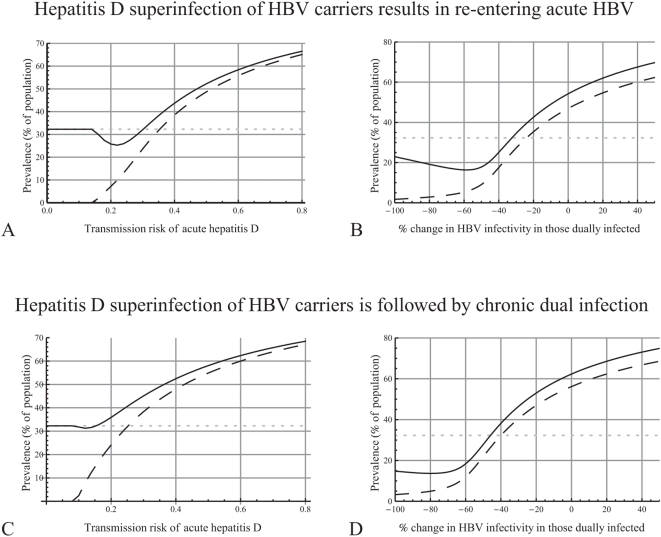
How the characteristics of hepatitis D affect the endemic prevalences of hepatitis B (HBV) and hepatitis D. The plot shows the prevalences of HBV (solid line) and hepatitis D (dashed line) in a population where both viruses are circulating and the prevalence of HBV in a population where only HBV is circulating (grey dotted line). A. The transmission probability of acute hepatitis D (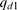) was varied from 0 to 0.8 and that of chronic hepatitis D (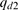)was 0.65 times that of acute hepatitis D. B. The percentage change in HBV infectivity in those dually infected (compared to those with single HBV infection, 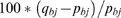, for *j* = 1, 2) was varied from −100% to +50%. C, D. As in plots A, B, but assuming that HBV carriers who are superinfected with hepatitis D do not re-enter acute HBV (using equations (2) instead of the last two equations of system (1)).

The endemic prevalences of HBV and hepatitis D are shown in Fig. 4A for a range of values of hepatitis D infectivity. For comparison, we also calculated the endemic prevalence of HBV for a hypothetical scenario where hepatitis D has not been introduced in the population and only HBV is circulating. The parameter values used relate to sexual transmission among men having sex with men (see Table 1 and Text S1). If the transmissibility of hepatitis D is very low, hepatitis D cannot be sustained in the population and only HBV remains endemic. If hepatitis D infectivity is sufficiently high, then both viruses remain endemic and the prevalence of hepatitis D increases as its infectivity increases. Comparing an epidemic where only HBV is circulating (grey dotted line) with an epidemic where both HBV and hepatitis D are circulating (black lines), and keeping the properties of HBV otherwise equal, the following observations can be made:

- If hepatitis D infectivity is not very high, the presence of hepatitis D results in lower endemic HBV prevalence. This can be understood intuitively, because now many individuals have dual infection and transmit HBV less than individuals with single HBV, resulting in less prevalent HBV infections.
- If hepatitis D infectivity is high, then hepatitis D spreads very fast and, hence, despite the lower transmissibility of HBV in those dually infected, those with single HBV infection are superinfected sooner rather than later with hepatitis D and, hence, frequently during acute infection. Since the probability of entering the chronic phase for those with acute dual infection is five times higher than for those with acute single infections, this results in higher endemic HBV prevalence and, thus, in a more severe HBV epidemic.

#### Suppression of HBV replication due to hepatitis D

The above results indicate that the suppression of HBV replication and transmissibility due to hepatitis D infection in those dually infected has an important role in the spread of the two viruses. To investigate this further, the endemic prevalences of HBV and hepatitis D were again calculated, but now for different levels of suppression of HBV replication (Fig. 4B). If hepatitis D reduces sufficiently the infectivity of HBV in those dually infected, the presence of hepatitis D results in lower endemic HBV prevalence and hence makes the HBV epidemic less severe. Otherwise, HBV prevalence is higher than in the absence of hepatitis D, which means that hepatitis D makes the HBV epidemic more severe.

#### Hepatitis D superinfection does not lead to acute HBV

Further we investigated the assumption that HBV carriers who are superinfected with hepatitis D go again through acute HBV infection. Fig. 4C,D show the endemic prevalences of HBV and hepatitis D, as in Fig. 4A,B, but assuming that HBV carriers who are superinfected with hepatitis D go directly to chronic dual infection. In this case, the last two of the model equations (1) are substituted by the following equations:
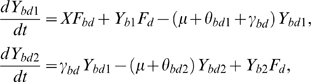
(2)This modification has two contradicting effects: (i) those superinfected with hepatitis D are in total less infectious (because they do not go through the acute phase); hence we would expect less transmission of HBV and of hepatitis D; (ii) those superinfected with hepatitis D now progress to chronic infection, while only a fraction of them will progress from acute to chronic, if superinfection leads to acute infection first; therefore we would expect higher prevalence and higher transmission of HBV and of hepatitis D. Fig. 4 shows that if carriers who are superinfected do not re-enter the acute stage, the prevalence of hepatitis D is higher and the virus remains endemic with lower infectivity. Also, the prevalence of HBV is higher, even with lower hepatitis D infectivity, since all superinfections become chronic infections and there are no infections “lost” due to recovery from acute infection, as explained also in Fig. 4A. This effect of hepatitis D on HBV is diminished, resulting in lower HBV prevalence, only if hepatitis D suppresses HBV infectivity too much in those dually infected (for instance, more than 65% with the parameters examined here), since then they can cause only too few new infections. Therefore, the assumption that hepatitis D superinfection causes HBV carriers to go again through the acute HBV stage results in a milder epidemic than if they would directly progress to chronic dual infection.

### The impact of control measures for HBV

**Figure 5:**
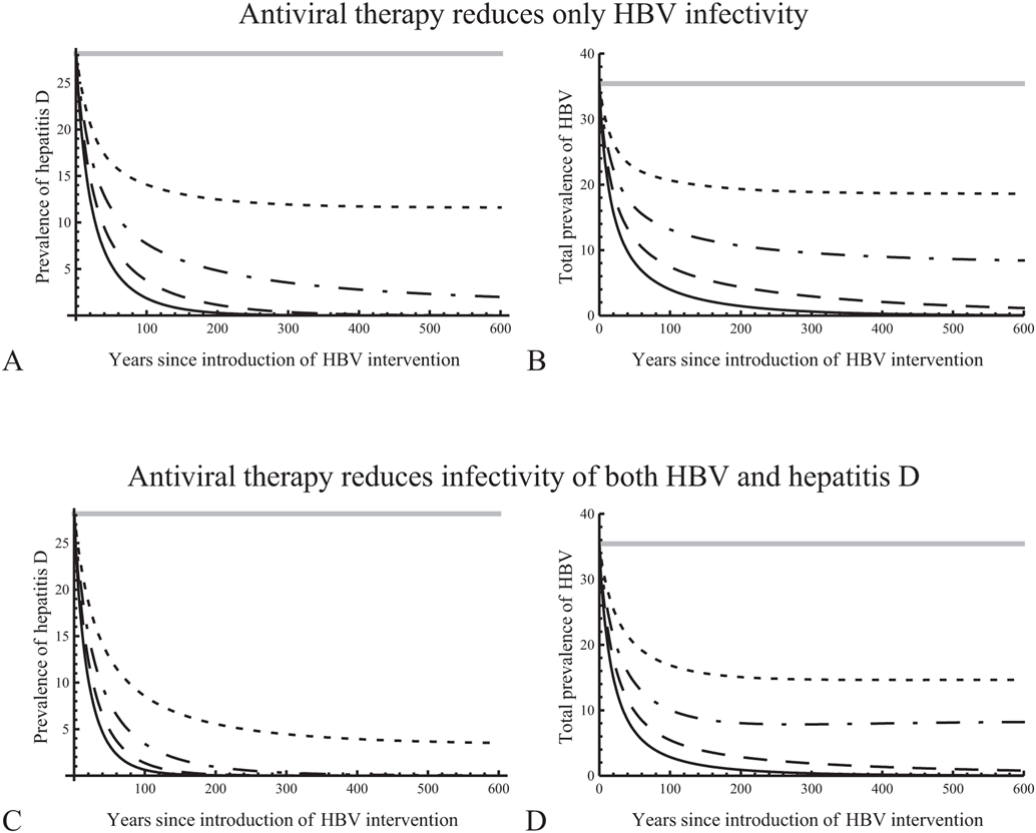
How antiviral treatment reduces the prevalences of hepatitis B (HBV) and hepatitis D in time. A, B. Treatment reduces only the infectivity of HBV. It is introduced at time 0, after the epidemic had stabilised at the endemic equilibrium with both viruses present, which is shown with a straight grey line. The infectivity of chronic HBV is reduced by 20% (dotted lines), 30% (dashed-dotted lines), 40% (dashed lines), or 50% (solid lines). Prevalences are shown as percentages of total population. C, D. As in plots A, B, but assuming that treatment reduces also the infectivity of hepatitis D, by the same percentage as that of HBV.

Here we examine the effect of control measures, such as treatment, reducing the transmissibility of chronic HBV equally in those with single (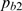) and those with dual infections (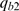). Since antiviral agents have no effect on hepatitis D [9], the model accounts for no reduction in infectivity of hepatitis D due to HBV treatment. The intervention was introduced when the epidemic had stabilised at the endemic equilibrium with both viruses prevalent (with the parameters as shown in Table 1, where HBV prevalence is slightly higher than what it would have been in the absence of hepatitis D). By reducing the infectivity of HBV alone, reductions in both HBV and hepatitis D can be achieved (Fig. 5A,B). With the highest reductions in infectivity shown here (40% and 50%), both viruses are eliminated; hepatitis D is eliminated much earlier than HBV. For completeness, we repeated the plots in Fig. 5A,B, assuming that treatment reduces also the infectivity of hepatitis D (Fig. 5C,D). In this case, the prevalence of hepatitis D declines much faster and the virus is eradicated much earlier or it stabilizes at a lower endemic prevalence (in the cases where it remains endemic). Also, the prevalence of HBV is slightly lower.

Figure 2 shows how much HBV infectivity should be reduced in order to eradicate HBV and hepatitis D. If hepatitis D suppresses HBV replication (Fig. 2A,B), the control of HBV is not affected by the presence of hepatitis D: HBV is eradicated with the same reduction in HBV infectivity in both epidemics (with and without hepatitis D). However, the resulting endemic prevalence is lower than that in an epidemic without hepatitis D. In this case, hepatitis D helps limiting the spread of HBV and enhances the impact of the intervention. On the other hand, if HBV replication is not suppressed by hepatitis D (Fig. 2C,D), higher reductions in HBV infectivity are required to eradicate the viruses. Also, the resulting endemic prevalence is higher than that in an epidemic without hepatitis D. In this case, the presence of hepatitis D makes the control of HBV more difficult and makes the interventions less effective.

**Figure 6:**
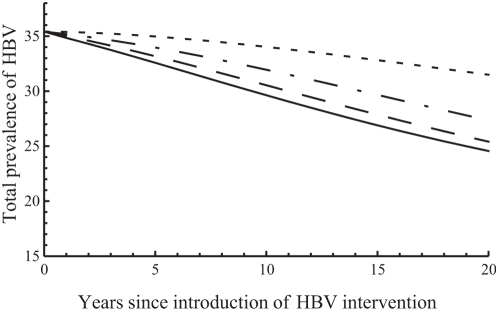
The initial impact of treatment on the prevalence of hepatitis B (HBV). Antiviral treatment reduces infectivity of chronic HBV by 20%. The total prevalence of HBV is shown as percentage of the population. When the intervention is introduced (year 0), 36% of the population is infected with HBV; among them, the percentage coinfected with hepatitis D is 30% (solid line), 20% (dashed line), 10% (dashed-dotted line), or 0% (dotted line).

In many countries, surveillance of hepatitis D is limited and therefore the actual prevalence of hepatitis D in the population is unknown. For that reason, we examined the impact of a specific intervention for HBV under different assumptions about the “unknown” prevalence of hepatitis D when the intervention was introduced, but keeping the total prevalence of HBV constant (Fig. 6). The intervention examined here is the reduction of the transmission probabilities of chronic HBV by 20% for those with single or dual infection. With higher hepatitis D prevalence at the time the intervention is introduced, HBV prevalence is reduced more, which means that in the beginning the intervention has a higher impact. This can be explained by the fact that for those with single HBV infection, infectivity is reduced by 20% due to treatment, while for those with dual infection, it is reduced by 20% on top of the 30% reduction due to suppression of HBV replication. Therefore, the more individuals with dual infection are present in the beginning (meaning, the higher the initial hepatitis D prevalence), the higher the total reduction in HBV infectivity and consequently the lower the prevalence of HBV. In time, however, as hepatitis D prevalence declines due to treatment, the effect of HBV replication also declines, and HBV prevalence is finally lower without hepatitis D than in the presence of hepatitis D.

## Discussion

The results presented in this study indicate that the presence of hepatitis D may have a strong impact on the spread of HBV. Hepatitis D virus modulates both the severity of the HBV epidemic and the impact of interventions that are aimed at reducing HBV incidence. The presence of hepatitis D virus may hamper the eradication of HBV. Interventions that aim to reduce the basic reproduction number of HBV below one may not be sufficient to eradicate the virus, as control of HBV depends also on the reproduction numbers of dual infections. This implies that for populations where hepatitis D is endemic, plans for control programs ignoring the presence of hepatitis D may underestimate the HBV epidemic and produce overoptimistic results.

The impact of hepatitis D can be explained as follows. At the individual level, hepatitis D affects HBV infection in two ways: HBV transmission rate is lower and the chance to progress from acute to chronic infection (and not recover) is higher for those with dual infection than for those with single HBV infection. Because of this, hepatitis D superinfection affects the prevalence of HBV at the population level. If hepatitis D infectivity is high, then hepatitis D superinfection will usually occur early during acute HBV infection; that increases the chance to progress to chronic infection and hence the prevalence of HBV. If hepatitis D infectivity is low, then hepatitis D superinfection will mostly occur during chronic HBV infection; that reduces HBV infectivity and hence HBV prevalence. The precise mechanism depends on how much HBV infectivity is reduced by hepatitis D, how much the progression rate to chronic infection is increased by hepatitis D, and by other properties of the two viruses. Our results, for instance, show that if hepatitis D superinfection does not result in re-entering the acute stage, higher HBV prevalence will be observed even with low hepatitis D transmission rates. Unfortunately, knowledge about the properties of dual infection is limited, as there are few studies on hepatitis D. The use of a mathematical model allows us to incorporate existing information and obtain realistic parameter values from the literature; the uncertainty analysis of the model further shows how the outcomes depend on the specific parameter values.

This study follows a considerable amount of research on the epidemic dynamics of interacting pathogen strains (see, for instance, [10]–[12]) and interacting pathogens (see, e.g., [13], [14]). Our results relate to those of other co-infections, for instance with HIV and tuberculosis; the observations show that incidence of tuberculosis has increased due to increased prevalence of HIV infection [15], [16]. The mechanism is that HIV impairs host immunity and substantially alters the infection dynamics of tuberculosis. Modeling studies have shown synergistic effects and that antiviral treatment for HIV is necessary for the reduction of tuberculosis prevalence [17], [15]. However, the system studied here is different in that hepatitis D is not a true pathogenic virus but rather a subviral agent incapable of disseminating without help from HBV. The requirement for a helper virus is a rare property among human viruses. Only one other helper-dependent infectious agent of humans is known (the adeno-associated virus, a parvovirus that requires adenovirus as helper [18]). Although the dynamics of a defective virus with a helper virus are reminiscent of those of synergistic co-infection, they are essentially different in that, whereas endemic equilibria of both tuberculosis without HIV and HIV without tuberculosis are possible under specific conditions, biology dictates that an endemic equilibrium with only the defective virus but not the helper virus is impossible under any circumstance.

The work presented here can be broadened towards several interesting research directions. For instance, the model can be extended to account for the effect of HBV vaccination. As yet, there are no effective treatments specific for the hepatitis D component of concurrent HBV and hepatitis D infections. However, vaccination against HBV provides direct protection against hepatitis D virus infection as well. In the recent years, HBV vaccination has been introduced in many countries, resulting in considerable reductions in HBV prevalence and incidence. Moreover, vaccination reduces HBV transmission and, hence, the number of those infected with HBV. As hepatitis D can be transmitted only in the presence of HBV, the hepatitis D prevalence will also decline. Our results for the treatment of HBV infections lead us to expect that vaccination may also have a large impact on the prevalence of hepatitis D. This expectation is confirmed by observations in countries where HBV vaccination is introduced and where hepatitis D is prevalent. For instance, in Taiwan the national HBV vaccination of infants was introduced in 1984; 15 years later, the HBsAg carrier rate in children had decreased from 9.8% to 0.7% [6]. The prevalence of hepatitis D among Taiwanese drug users infected with HBV, the most important risk group for hepatitis D transmission, decreased from 91% in 1986 to 39% in 1997 [6].

Further, it would be also interesting to account for stochasticity in this framework. Several studies have shown that including chance in the model can change its long-term behaviour, for instance “fit” pathogens that remain endemic in the deterministic model, may go extinct in the stochastic due to chance fading out [19], [20]. In models of multiple pathogen strains, it has been shown that in cases where the deterministic model predicts the coexistence of multiple strains, the stochastic model predicts the extinction of one or all strains [21], [22]. It is possible that a stochastic analysis of the dynamics of HBV and hepatitis D would also alter the results about the coexistence of the two viruses; this is an area where future research should definitely receive more attention.

An important direction for further work would be to include spatial structure in the model. The assumption of proportionate mixing in a large population ignores the clustering of individuals at high risk. As both HBV and hepatitis D spread through the same transmission routes, it is likely that there are clusters of individuals with a high prevalence of HBV, where hepatitis D can disappear by chance. To study the dynamics of such a system, we require a meta-population model or a network model. It has been shown that the dynamics of infection in such a network may differ, for instance having less opportunity for persistence of the disease, or greater possibility for extinction and limit cycles (see, e.g. [23], [24]). Our analysis presents a mean-field model of such a more complex model and should be understood as a first step towards understanding the complicated dynamics of interaction between hepatitis D and HBV infection dynamics.

Another issue that could be investigated in further research is the transmission of the two viruses via other contacts. In several countries injecting drugs and household contacts are also important routes of transmission of both HBV and hepatitis D [6], [7], [25]. It is important to examine whether (and how) the dynamics of the two viruses differ according to the route of transmission and the risk groups in which they are prevalent. Also, a number of studies have indicated that certain properties of hepatitis D infection may be different in those superinfected with hepatitis D compared to those who were infected with both viruses at the same time (for instance, different progression rates [2]). The model could be adapted to account for such differences, if those dually infected are divided into two types, those superinfected and those coinfected. Finally, the model can be extended to include a separate subgroup of the population for those recovering from the infection and becoming immune.

Finally, certain limitations of this modeling study have to be mentioned. First, because of the limited knowledge on the biological properties of hepatitis D and on how HBV infection is changed in those dually infected, several assumptions were made in the model structure or the parameter values used in the numerical results. We tried to compensate the lack of data by performing uncertainty analyses and examining different scenarios, covering as much as possible of all realistic possibilities. Another consideration is that we did not account for variation in some progression rates of HBV according to age. This was done because these rates are relatively stable for adults during the years of sexual activity examined in the numerical results [26], [27]. Therefore, it is expected that stratifying by age is not necessary and would not affect considerably the results. Finally, in the present study it was assumed that individuals recovering from HBV or hepatitis D infection and those developing severe complications are removed from the population and do not contribute further to the transmission of the two viruses. Actually, those recovering become immune, remain in the population, and may have contacts with those not immune. Therefore, the total population size is underestimated in the model and the incidence is overestimated. However, this holds for both viruses and we expect that it does not considerably affect the balance between the two viruses or their interaction. This simplification allows us to sketch the expected qualitative dynamic behavior and the potential impact that hepatitis D has on the spread and control of HBV.

To our knowledge, this is the first attempt to model the dynamics of hepatitis D and to investigate the interplay between HBV and hepatitis D. On the empirical side, there is very little known about the epidemiological properties of hepatitis D, and given its importance in affecting the HBV epidemic, more information is needed on basic epidemiological characteristics of hepatitis D infection and the time course of infection with HBV and a concurrent hepatitis D infection. On the theoretical side, understanding the complex dynamics of the interaction between a defective virus and its helper virus would be much helped by additional modeling approaches that incorporate the role of demographic stochasticity and network models. Our findings indicate that hepatitis D plays an important role in the spread and control of HBV. Investigating the transmission dynamics of HBV should account for the presence of hepatitis D in a population. Augmenting the existing HBV monitoring programs with monitoring of hepatitis D could boost the accuracy of the surveillance of HBV prevalence and of the efficacy of control programs.

## Supporting Information

Text S1 (0.04 MB PDF)
