# Supplementary material for: How Hepatitis D Virus Can Hinder the Control of Hepatitis B Virus
Source: PLoS One. 2009 Apr 21;4(4):e5247. doi: 10.1371/journal.pone.0005247 (PMC2668760; doi:10.1371/journal.pone.0005247)
Supplement: Text S1 — (0.04 MB PDF) [file pone.0005247.s001.pdf]

How Hepatitis D Virus Can Hinder the Control of Hepatitis B Virus

Xiridou M, Borkent-Raven B, Hulshof J, Wallinga J

Supporting Information: Text S1

Note: ther references in this document are not as in the main text; see end of Text S1 for references.

The invasion reproduction number

Let X^* be the number of uninfected individuals and Y_{b1}^*, Y_{b2}^* the numbers of HBV infected individuals at the endemic state with only HBV. Then

$$\frac{X^*}{N^*} = \frac{\mu + \theta_{b1} + \gamma_b}{\phi(p_{b1} + \frac{\gamma_b p_{b2}}{\mu+\theta_{b2}})}, \quad Y_{b1}^* = \frac{B - \mu X^*}{\mu + \theta_{b1} + \gamma_b}, \quad Y_{b2}^* = \frac{\gamma_b}{\mu + \theta_{b2}} Y_{b1}^*, \qquad \text{(A.1)}$$

$$\text{where} \qquad N^* = \frac{B\pi_3}{1 - (1 - \mu\pi_3)/R_b}, \qquad \pi_3 = \frac{\mu + \gamma_b + \theta_{b2}}{(\mu + \gamma_b + \theta_{b1})(\mu + \theta_{b2})}.$$

The invasion reproduction number, $\hat{R}_{bd}$, can be interpreted as follows. The duration of time that an individual with dual infection stays in the first stage of his infection is $1/(\mu + \theta_{bd1} + \gamma_{bd})$. During this time he has $\phi/(\mu + \theta_{bd1} + \gamma_{bd})$ contacts. A fraction X^*/N^* of these contacts are with people that are not infected with HBV yet. The probability of transmission of both HBV and hepatitis D is then $q_{b1}q_{d1}$. This gives

$$\frac{X^*}{N^*}\frac{\phi q_{b1} q_{d1}}{\mu + \theta_{bd1} + \gamma_{bd}}$$

new infections with both HBV and hepatitis D. The other contacts will be with persons that are already infected with HBV, resulting in

$$\frac{Y_{b1}^* + Y_{b2}^*}{N^*}\frac{\phi q_{d1}}{\mu + \theta_{bd1} + \gamma_{bd}}$$

new cases. An individual with dual infection progresses to stage 2 at a rate $\gamma_{bd}/(\mu + \theta_{bd1} + \gamma_{bd})$. During the second stage, he produces

$$\frac{\phi q_{d2}}{\mu + \theta_{bd2}}\frac{q_{b2}X^* + Y_{b1}^* + Y_{b2}^*}{N^*}$$

new infections. Adding these, it results that

$$\hat{R}_{bd} = \frac{\phi}{\mu + \theta_{bd1} + \gamma_{bd}} \left(q_{d1} \frac{q_{b1}X^* + Y_{b1}^* + Y_{b2}^*}{N^*} + \frac{\gamma_{bd} q_{d2}}{\mu + \theta_{bd2}} \frac{q_{b2}X^* + Y_{b1}^* + Y_{b2}^*}{N^*} \right).$$

Substituting from equation (A.1) and from the definitions of R_b and R_{bd}, the equation for $\hat{R}_{bd}$ is obtained. Notice that $\hat{R}_{bd}$ is defined when the endemic equilibrium with only HBV exists and that is when $R_b > 1$. In this case, $\hat{R}_{bd} > 0$ and $\hat{R}_{bd} > R_{bd}$.

Stability of equilibria

The Jacobian of the disease-free equilibrium is a block diagonal matrix, with the following matrices on the diagonal

$$U_0 = \begin{pmatrix} -\mu & -\phi p_{b1} & -\phi p_{b2} \\ 0 & -w_b + \phi p_{b1} & \phi p_{b2} \\ 0 & \gamma_b & -\mu - \theta_b \end{pmatrix}$$

$$\text{and} \qquad V_0 = \begin{pmatrix} -w_{bd} + \phi q_{b1} q_{d1} & \phi q_{b2} q_{d2} \\ \gamma_{bd} & -\mu - \theta_{bd} \end{pmatrix},$$

where $w_b = \mu + \theta_{b1} + \gamma_b$ and $w_{bd} = \mu + \theta_{bd1} + \gamma_{bd}$. It can be easily shown that the eigenvalues of U_0 are negative if $R_b < 1$ and the eigenvalues of V_0 are negative if $R_{bd} < 1$. If one of the R_b and R_{bd} is larger than one, then one of the eigenvalues of U_0 or V_0 is positive. Hence, the disease-free equilibrium is stable if and only if $R_b < 1$ and $R_{bd} < 1$.

The Jacobian of the equilibrium with HBV only is also block triangular with the matrices U_b and V_b on the diagonal, where

$$U_b = \begin{pmatrix} -\mu - G_b\left(1 - \frac{1}{R_b}\right) & -\frac{1}{R_b}(-G_b + \phi p_{b1}) & -\frac{1}{R_b}(-G_b + \phi p_{b2}) \\ G_b\left(1 - \frac{1}{R_b}\right) & \frac{1}{R_b}(-G_b + \phi p_{b1}) - w_b & \frac{1}{R_b}(-G_b + \phi p_{b2}) \\ 0 & \gamma_b & -(\mu + \theta_{b2}) \end{pmatrix},$$

$$\text{and} \qquad V_b = \begin{pmatrix} -w_{bd} + \phi q_{d1}\left[\frac{1}{R_b} q_{b1} + \left(1 - \frac{1}{R_b}\right)\right] & \phi q_{d2}\left[\frac{1}{R_b} q_{b2} + \left(1 - \frac{1}{R_b}\right)\right] \\ \gamma_{bd} & -\mu - \theta_{bd} \end{pmatrix}.$$

After some calculations, it can be shown that the eigenvalues of U_b are negative if $R_b > 1$ and the eigenvalues of V_b are negative if $\hat{R}_{bd} < 1$. Hence, the equilibrium with only HBV is stable if $R_b > 1$ and $\hat{R}_{bd} < 1$, and unstable otherwise.

Parameter values

For HBV, acute infection lasts three months [1,2]. Among those with acute infection, 10% progresses to chronic infection [3,1], hence $\gamma_b = 0.10/0.25 = 0.4$ per year, and 90% recovers, which means $\theta_{b1} = 0.90/0.25 = 3.6$ per year. The recovery rate for carriers is 0.02 per year [2,4]. The per partnership transmission probability from an individual infected with HBV is $p_{b1} = 0.46$ during acute and $p_{b2} = 0.30$ during chronic infection [5,4]. The relative infectivities of chronic versus acute infection were kept at $0.30/0.46 = 0.65$, throughout all calculations, for both HBV and hepatitis D.

For hepatitis D, unfortunately little is known and therefore many assumptions had to be made. The uncertainty in the values of some parameters was examined as explained in the results. hepatitis D superinfection of an HBV carrier causes a generally severe acute hepatitis that leads to chronic hepatitis in up to 80% of cases [6]. Serologic data of a cohort of Indians who developed hepatitis suggested that most dual infections were due to hepatitis D superinfection of HBV carriers and that more than 60% of these cases progressed to chronic disease [7]. Therefore, we assumed that among dual infections, 50% of acutes progresses to chronic infection and 50% recovers. No data indicate a difference in the duration of acute infection between those dually infected and those with single HBV infection. Therefore, $\gamma_{bd} = 0.50/0.25 = 2$ and $\theta_{bd1} = 0.50/0.25 = 2$ per year. For HBV carriers, it has been reported that the rate of positivity for HBV DNA is lower in those coinfected with hepatitis D than in those negative for hepatitis D infection: 52% to 73% respectively [8]. Therefore we assumed that HBV infectivity for those dually infected is $52/73 = 0.71$ times that for individuals with single HBV infection ($q_{bj} = 0.71 p_{bj}$, $j = 1, 2$). For the transmission probabilities of hepatitis D, no estimates were found in the literature, thus they were taken equal to those of HBV for persons with dual infection ($q_{dj} = q_{bj}$) and other values were examined in the uncertainty analysis.

Initially, the total population size is $n = 26000$ [9]. The rate of departing from the population is $\mu = 0.018$ per year, assuming that men are sexually active for 55 years (ages 15–70 years old). Uninfected individuals enter the population at a rate $B = \mu n$, to account for a constant population size in the absence of the infections.

REFERENCES

1. Zhao S, Xu Z, Lu Y (2000) A mathematical model of hepatitis B virus transmission and its application for vaccination strategy in China. Intern J Epid 29: 744–752.
2. Edmunds W, Medley G, Nokes D (1996) The transmission dynamics and control of hepatitis B virus in The Gambia. Stat Med 15: 2215–33.
3. WHO (2002) Hepatitis B. Available at: http://www.who.int/csr/disease/hepatitis/HepatitisB-whocdscsrlyo2002-2.pdf. Accessed 2008 June 4.
4. Mast E, Mahoney F, Kane M, Margolis H (2004) Hepatitis B vaccine. In: Plotkin S, Orenstein W, editors. Vaccines. WB Saunders, Philadelphia, pp. 299–337. 4th ed.
5. Williams J, Nokes D, Medley G, Anderson R (1996) The transmission dynamics of hepatitis B in the UK: a mathematical model for evaluating costs and effectiveness of immunization programmes. Epidemiol Infect 116: 71–89.
6. WHO (2001) Hepatitis Delta. Available at: http://www.who.int/csr/disease/hepatitis/HepatitisD-whocdscsrncs2001-1.pdf. Accessed 2008 June 4.
7. Hadler S, De Monzon M, Ponzetto A, Anzola E, Rivero D, et al. (1984) Delta virus infetion and severe hepatitis. An epidemic in the Yucpa Indians of Venezuela. Ann Intern Med 100: 339–344.
8. Kao J, Chen P, Lai M, Chen D (2002) Hepatitis D virus genotypes in intravenous drug users in Taiwan: decreasing prevalence and lack of correlation with hepatitis B virus genotypes. J Clin Microb 40: 3047–9.
9. van Houdt R, Sonder G, Dukers N, Bovee L, van den Hoek A, et al. (2007) Impact of a targeted hepatitis B vaccination program in Amsterdam, The Netherlands. Vaccine 25: 2698–705.
